# ParBiBit: Parallel tool for binary biclustering on modern distributed-memory systems

**DOI:** 10.1371/journal.pone.0194361

**Published:** 2018-04-02

**Authors:** Jorge González-Domínguez, Roberto R. Expósito

**Affiliations:** Grupo de Arquitectura de Computadores, Universidade da Coruña, A Coruña, Spain; Mayo Clinic Arizona, UNITED STATES

## Abstract

Biclustering techniques are gaining attention in the analysis of large-scale datasets as they identify two-dimensional submatrices where both rows and columns are correlated. In this work we present *ParBiBit*, a parallel tool to accelerate the search of interesting biclusters on binary datasets, which are very popular on different fields such as genetics, marketing or text mining. It is based on the state-of-the-art sequential Java tool *BiBit*, which has been proved accurate by several studies, especially on scenarios that result on many large biclusters. *ParBiBit* uses the same methodology as *BiBit* (grouping the binary information into patterns) and provides the same results. Nevertheless, our tool significantly improves performance thanks to an efficient implementation based on C++11 that includes support for threads and MPI processes in order to exploit the compute capabilities of modern distributed-memory systems, which provide several multicore CPU nodes interconnected through a network. Our performance evaluation with 18 representative input datasets on two different eight-node systems shows that our tool is significantly faster than the original *BiBit*. Source code in C++ and MPI running on Linux systems as well as a reference manual are available at https://sourceforge.net/projects/parbibit/.

## Introduction

The amount of data that can be collected and stored in several research and industry fields has significantly increased during the last years. This information is often described in a two-dimensional way, where rows and columns represent the measured attributes and samples, respectively. The analysis of these data is a complex and computationally expensive procedure that often requires data mining techniques to extract valuable information and transform it into an understandable structure for further use. A widely spread data mining approach is the clustering, that allows to identify some patterns and structures between the attribute and sample relationships. However, traditional clustering techniques are not able to provide information to understand local relationships between both samples and attributes. In this case, biclustering approaches should be used in order to identify a subset of rows (attributes) that exhibit similar patterns on a subset of columns (samples) in a two-dimensional data matrix.

There exist several biclustering techniques, with different advantages and drawbacks depending on the characteristics of the input datasets and the research field where the approach will be used [1]. Gene expression analyses are probably nowadays the most common application of biclustering techniques. In this case datasets contain information about the expression level of many genes on several individuals under different experimental conditions. Rows and columns represent genes and samples, respectively. Biclustering is able to diagnose genes responsible of certain diseases only on a group of individuals. Many research works have focused on analyzing the best biclustering approaches for gene expression data [2–4]. Other fields where biclustering has been satisfactorily applied are drug activity [5], text mining [6], information theory [7] or marketing [8].

Among the different alternatives for biclustering, some algorithms are especially designed for binary data so that they are able to obtain results with better accuracy in lower runtime over these very common datasets. For instance, in genetics the data can be simplified so that each value one or zero represents whether a gene is differentially expressed in an individual or not. A recent survey has proved that binary biclustering can provide high precision results for gene expression data analyses [9]. Binary data matrices are also useful in text mining (values are one only when certain word appears in a document) or marketing (each value represents whether a costumer buys a product or not).

Despite binary biclustering techniques are usually faster than those for quantitative data, the computational cost of the available methods that provide accurate results is still prohibitive for large datasets. This paper presents *ParBiBit*, a parallel application that exploits computational capability of modern distributed-memory systems to accelerate the search of biclusters on binary datasets. It is implemented with a hybrid approach that uses MPI [10] to work on different nodes connected through a network, with the multithreaded support of C++11 [11] to exploit several cores within the same node.

## Related work

Several biclustering approaches have been suggested to deal with binary two-dimensional matrices. Among all of them, we have selected the Java-based application *BiBit* [12] as basis for our tool due to several reasons:

- A recent review of 17 available biclustering methods [9] has proved that *BiBit* obtains accurate results for gene expression data, especially on cases with many large biclusters. This work also shows that the *BiBit* approach can be useful for quantitative data if applying a binarization.
- *BiBit* exploits the binary nature of the data by efficiently using Boolean algebra operations. This makes it faster than *Bimax* [13], probably the most commonly employed binary biclustering tool.
- Although in the last years several algorithms not tested in the aforementioned review have been presented for binary biclustering [14–16], their related publications do not include tests that prove that any of these novel approaches are more accurate than *BiBit*. Furthermore, these implementations are not publicly available for further testing.

*ParBiBit* is significantly faster than *BiBit* thanks to an efficient C++ implementation and its ability to exploit the computational capabilities of large systems with several multicore nodes connected through a network (also known as CPU clusters). Previous works that address the biclustering on this type of facilities are available for quantitative datasets following either the message-passing paradigm [17, 18] or the MapReduce approach [19, 20]. The only work focused on binary data [21] is implemented with this last MapReduce paradigm. However, all these previous works seem preliminary implementations as their parallel software have not been released. Consequently, up to our knowledge, *ParBiBit* is the first publicly available tool to accelerate binary biclustering on multicore CPU clusters. Finally, implementations designed for other type of high performance computing architectures such as GPUs [22–24] or FPGAs [25, 26] have also been presented, but none of them dedicated to binary data.

## Background

This section describes the main concepts and technologies on which *ParBiBit* relies on, and thus are necessary to understand the behavior and implementation of our tool.

### Binary biclustering: The BiBit approach

A bicluster in a binary matrix *M* with dimensions *m* × *n* consists of a set of rows and columns (*R*, *C*) so that all the values within that subset are one. A formal definition can be: ∀*i* ∈ *R*, ∀*j* ∈ *C*, *M*[*i*, *j*] = 1. Additionally, most tools search for only maximal biclusters, i.e., those that are not entirely contained by any other bicluster.

Similarly to *BiBit*, our tool uses the concept of *bit-pattern* in order to find the biclusters of a binary matrix with a minimum number of rows (*mnr*) and columns (*mnc*) specified by the user. The joint pattern of a subset of rows consists of *n* bits (one per column) where the bit *k* is set to one if the binary value of column *k* in all the rows of the subset is equal to one. Otherwise, the bit is set to zero. It means that the joint pattern *p* of a subset of rows (*r*_1_, *r*_2_, …, *r*_*z*_) can be defined as: *p* = *r*_1_ ∧ *r*_2_ ∧ … ∧ *r*_*z*_, where ∧ is the binary AND operator. The pattern of a bicluster is the joint pattern of all the rows contained in it. *BiBit* works as follows (we refer to the *BiBit* main publication [12] for more details):

1. Initializes an empty list of bicluster structures.
2. For each pair of rows (*r*_*i*1_, *r*_*i*2_) from the input matrix *M*:
  - Creates a new bicluster with pattern *p* = *r*_*i*1_ ∧ *r*_*i*2_ and rows *r*_*i*1_, *r*_*i*2_.
  - Checks that the number of ones in the pattern is equal or higher than *mnc* and the pattern has not been used for any bicluster already inserted in the list. Otherwise, the bicluster is discarded and the algorithm returns to step 2.a for new pair.
  - All rows *r*_*i*3_ different than *r*_*i*1_ and *r*_*i*2_ are compared to *p*, and those that satisfy that *p* ∧ *r*_*i*3_ = *p* are included in the bicluster.
  - The bicluster is inserted in the list if the number of rows is equal or higher than *mnr*.
3. The information of the list is printed in the output file. The rows that belong to each bicluster were directly saved in the structure, while the columns can be obtained as those elements in the pattern equal to one.

However, the dependencies among the iterations of the loop in step 2 (each iteration checks whether the pattern has already been used) make the *BiBit* algorithm not adequate for parallel computing. Thus, it had to be modified in *ParBiBit* as will be explained in following sections.

### Multithreading with C++11

Historically, there have been several C and C++-based libraries that support multithreading over several CPU cores that share memory. Some examples are POSIX Threads [27] or Intel’s Threading Building Blocks [28]. This heterogeneous software landscape made it difficult to write platform-portable C/C++ codes. With the release of C++11 [11] and its novel multithreading API it is finally possible to write platform-independent code in C++ that is supported by compilers from both the Linux/UNIX world and the Windows ecosystem without the need for third party libraries.

When a C++ program is executed, only one main thread exists. An arbitrary number of software threads can be spawned by the main thread of a system process, and are represented in C++11 with an object of class *thread*. It is even possible to recursively spawn threads from within already spawned ones. The actual number of concurrently running threads should be adjusted to roughly match the amount of physical cores of the system since the OS might serialize their execution using expensive context switches if their number exceeds the amount of available cores. All threads share the resources of the parent system process, i.e., they can access the same memory space. This is advantageous since threads can be spawned with low latency and benefit from lightweight inter-thread communication using shared registers and arrays. The instruction flow of the main thread continues independently of the work accomplished in the spawned threads until the end of the main function is reached. In order to ensure that all spawned threads have finished their work, the main thread should wait for them. This is accomplished with a call to the method *join* of the class *thread*.

As all threads share the same memory space, one of the most common causes of errors are the race conditions, i.e., situations where two or more threads want to access the same data and they try to change it simultaneously. As the thread scheduling algorithm can swap between threads at any time, we do not know the order in which the threads will attempt to access the shared data. Therefore, the result of the change in data would be dependent on the thread scheduling algorithm, i.e., both threads are racing to access/change the data. The solution in C++11 consists in using an object of the class *mutex* to restrict the execution of critical sections to a certain thread in a mutually exclusive manner. A mutex can be locked by a specific thread, i.e., the subsequent code fragment can only be executed by the issuing thread until the mutex is released. While being locked a mutex cannot be locked or unlocked by other threads which have to wait for its release causing an implicit synchronization of threads. Nevertheless, it is not advisable to abuse of the use of mutexes as its synchronization leads to some performance overhead (some threads stop their execution and remain idle). In *ParBiBit*, mutexes are used to serialize the modification of the list of biclusters to ensure that all threads have the most updated information when they check whether the pattern has already been used (see point 2.d in the background subsection that describes the *BiBit* approach).

### Message passing interface (MPI)

**Fig 1:**
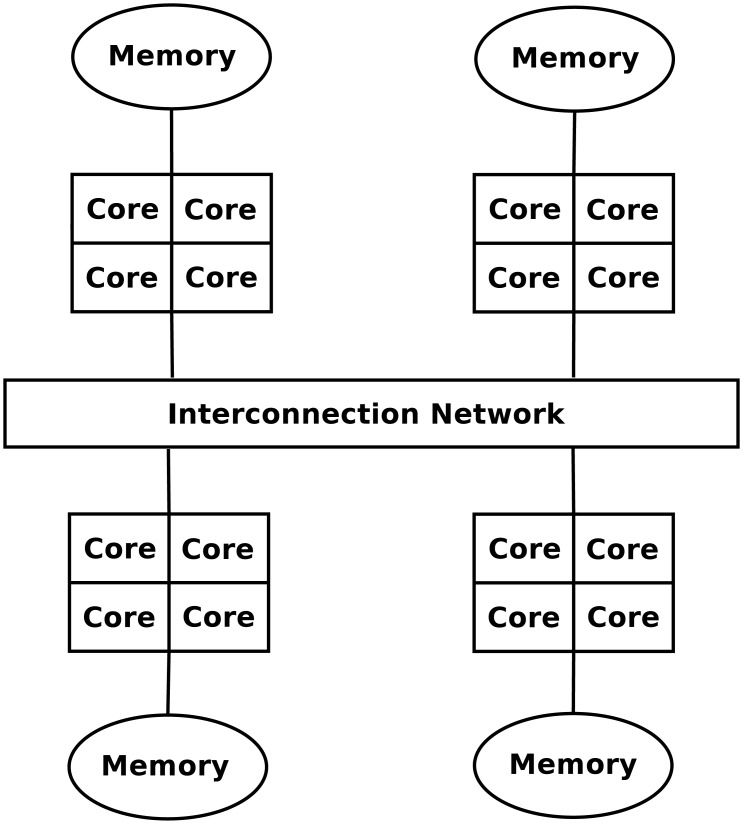
Abstraction of a distributed-memory system with several cores and one memory module per node.

The target parallel architecture of this work are distributed-memory systems that consist of several nodes interconnected through a network, each of them with a memory module and several CPU cores (see Fig 1). Parallel computing on this kind of systems usually follows the Single Program Multiple Data (SPMD) style, i.e., it splits the workload into different tasks that are executed on multiple CPUs so that all nodes and cores collaborate to accelerate computation. The computational capability of the cluster depends on factors such as the number of nodes, the number of cores per node, the network characteristics, the memory bandwidth, etc.

The most common programming model for high-performance cluster systems is message-passing. MPI [10] is established as a *de-facto* standard for message-passing as it is based on the consensus of more than 40 participating organizations, including vendors, researchers, software library developers, and users. MPI provides a portable, efficient, and flexible standard for message-passing. Note that it is only a definition of an interface, that has been implemented by several developers for different architectures. Nowadays there exist several implementations whose routines or functions can be directly called from C, C++, Fortran or Java code.

A parallel MPI program consists of several processes with associated local memory. In a pure MPI program each process is linked to one core. In hybrid MPI and multithreaded programs as *ParBiBit* each process is usually mapped to one node and it has several associated threads launched with the C++11 multithreading support (often the same number of threads as cores within the node). We should remark that each process has its own memory address space that cannot be directly accessed by other processes. If one process needs information stored in a remote memory module data communication must be performed, which is usually the main performance overhead. The traditional MPI communication style is two-sided, where the source and destination processes must be synchronized through either send and receive functions or collective routines for communications that involve more than two processes. Nevertheless, *ParBiBit* improves the efficiency of the internal data exchanges by making use of the Remote Memory Access (RMA) one-sided communications included in MPI since its 3.0 version. These kind of routines have been proved more efficient than two-sided communication on several scenarios, thanks to avoiding synchronizations between source and destination processes [29, 30].

## Methods

*ParBiBit* is a command line tool that receives as arguments some configuration parameters such as the path to the input and output files, the minimum number of rows (*mnr*) and columns (*mnc*) per bicluster, etc. An explanation of all the arguments, as well as installation and execution instructions, are included in the reference manual of the tool. Although *BiBit* introduces the bit-pattern approach used in our tool, its algorithm is not adequate for parallel computation. Therefore, instead of having one only loop with dependencies among all its iterations, *ParBiBit* divides the computation into the following phases:

1. Input reading. Read the two-dimensional data matrix with the input values for *m* attributes and *n* samples from a file with ARFF extension.
2. Binarization (optional). Although our tool is designed for binary biclustering, it also accepts real values in the input data file. In this case it applies a binarization procedure where all values higher than a certain threshold are set to one, and otherwise to zero. This threshold is also indicated as a parameter by command line. This approach has been used in previous analyses with satisfactory results [9].
3. Encoding. Instead of saving in memory a *m* × *n* matrix with the binary values, *ParBiBit* encodes the values associated to each attribute into an array of 32-bit integers with length $\frac{n}{32}$ (each integer contains the information of 32 samples). Working with encoded values accelerates the procedure of checking whether a row must be included in a bicluster (see point 2.c in the background subsection that explains the *BiBit* approach): for each 32 samples we only need one 32-bit AND operation, which is much faster than 32 1-bit AND operations. This encoding technique had already been applied in *BiBit* but with a 16-bit basis that is less effective than our 32-bit approach. The use of 64-bit (or even larger) encoded values was discarded as one 64-bit AND logic operation is not faster than two 32-bit AND operations on most computing platforms.
4. Bicluster initialization. Create one bicluster structure for each pair of rows with the following information: the joint pattern and the id of the two rows of the pair. Insert in a set all the structures that correspond to biclusters with different patterns.
5. Bicluster completion. For each bicluster with rows (*r*_*i*1_, *r*_*i*2_) available in the set from the previous phase, complete its information by checking whether all rows *r*_*i*3_ (with *i*3 ≠ *i*1, *i*2) belong to the bicluster. Include the id *i*3 in the structure when the condition is satisfied.
6. Output writing. The information of the biclusters found in the previous steps is written into an output file that follows the same format as for *BiBit*, i.e., one line per bicluster with the following values separated by semicolons:
  - An integer with the number of rows in the bicluster (*nr*).
  - An integer with the number of columns in the bicluster (*nc*).
  - A list of *nr* strings with the names or ids of the attributes included in the bicluster. They are explicitly stored in the structure.
  - A list of *nc* strings with the names or ids of the samples included in the bicluster. They can be obtained from the pattern, as those elements with bit one in the pattern represent columns included in the bicluster.

The impact of binarization and encoding on the total runtime is negligible, while the reading/writing of the input/output are I/O intensive phases without chances for parallelization. Therefore, we have focused on accelerating the phases that initialize and complete the biclusters (steps 4 and 5), which are the most computationally demanding ones (more than 98% of the total runtime when executing on one core). Finally, remark that the sequential C++ code of *ParBiBit* is more efficient than the *BiBit* one with Java, especially thanks to better memory management.

### Parallel bicluster initialization

Algorithm 1 shows the pseudocode of this phase, that receives as input the encoded data and the minimum number of columns, and whose goal is to provide a set of all the bicluster structures with only two row ids that have different patterns. The information of all these initialized biclusters will be extended in the next step. Each bicluster is represented as a structure with a list of attribute ids (integers) and a pattern (i.e., an array with $\frac{n}{32}$ 32-bit integers where a bit equal to one in position *j* represents that the value of the *j*-th sample is one in all the rows that belong to the bicluster). The C++ set container is used to save all bicluster structures as it works faster than a list for insertions, deletions and searches when each element can be identified by a unique key (logarithmic complexity instead of linear). In this case the key is equal to the pattern, as no biclusters with the same pattern are allowed. The C++ set is initially empty and is stored in shared memory so all threads can insert the structure and check whether the pattern is repeated.

**Algorithm 1:** Pseudocode of the multithreaded approach to initialize the biclusters.

1 INPUT: $m\times \frac{n}{32}$ 32-bit integer matrix *D* with the encoded data

2 INPUT: Integer *mnc* with the minimum number of columns per bicluster

3 Initialize empty bicluster set *S*

4 Initialize mutex *x*

5 # Multiple threads responsible of different *i* indexes

6 **for** *Each row i from* 0 *to m* − 2 **do**

7  **for** *Each row k from i* + 1 *to m* − 1 **do**

8   Initialize pattern *p* as empty array of 32-bit integers

9   Initialize *num*1 ≔ 0

10  **for** *Each encoded column j from* 0 *to* $\frac{n}{32}$ **do**

11   *p*[*j*] ≔ *D*[*i*][*j*] ∧ *D*[*k*][*j*]

12   *num*1 ≔ *num*1 + *popcount*(*p*[*j*])

    **end**

13  **if** *num*1 ≥ *mnc* **then**

14   Create bicluster structure *b* with *p*, *i* and *k*

15   Lock *x*

16   **if** *No bicluster structure with pattern p in S* **then**

17    Insert *b* in *S*

    **end**

18   Unlock *x*;

     **end**

   **end**

  **end**

Two loops that iterate among the rows of the encoded matrix *D* are necessary to work over all the pairs of attributes (Lines 6 and 7). The pattern of each pair is calculated by applying one 32-bit logical AND (∧) operation for each encoded value (Line 11). The number of ones in the pattern represents the number of samples that are included in the bicluster. Therefore, only those patterns with higher number of one values than *mnc* are useful (Line 13). The function popcount of Line 12 represents a custom-made routine that efficiently counts the number of positive bits of a 32-bit integer on a x86-based computing system.

The second condition that must be fulfilled in order to insert a new bicluster in the set is that no previous structure has the same pattern. Remark that several threads searching and/or inserting biclusters with the same pattern at the same time could lead to race conditions. A mutex is employed to serialize these accesses and thus avoid that two threads could simultaneously insert biclusters with the same pattern (Lines 15-18). In order to minimize the amount of serial work, the creation of the bicluster of Line 14 was removed from the critical section.

No MPI parallelization has been included in this step as its performance would not be satisfactory on distributed-memory nodes. Every time that one structure is initialized the process should check in the set whether the pattern is repeated. It could be performed either with a centralized container or with a copy of the set on each process. Nevertheless, both solutions would be extremely inefficient due to the large amount of MPI communications needed to synchronize each insertion in the set. Therefore, at the end of this phase only one process has the information available in its local memory.

### Bicluster completion

Bicluster completion consists in finding which rows belong to each of the already initialized biclusters. In this stage *ParBiBit* launches several MPI processes that work over different biclusters at the same time, applying a static distribution where the same amount of biclusters are assigned to each process. This distribution provides a well-balanced workload among processes as the computational cost of each bicluster completion is similar. Algorithm 2 illustrates the work performed by each process. It starts by copying the initial encoded data to the memory of all processes (Line 4), as this data is initially only available on the main process (the only one that worked during the previous phase) but will be needed by all of them. We use the MPI_Bcast collective that is usually faster than point-to-point communications [31, 32]. Although this data replication leads to memory overhead, it allows *ParBiBit* to reduce communication. In order to limit the memory overhead and make it affordable for current systems, *ParBiBit* does not create one MPI process per core (each one with its own copy of the encoded data). Instead, each process is related to a group of cores and launches several C++11 threads that are able to access shared memory, use the same copy of the data and collaborate to complete the biclusters assigned to their parent process. This hybrid model has already been satisfactorily applied to other fields such as bioinformatics [33], molecular dynamics [34] or linear algebra [35]. Our implementation is flexible enough to allow the users to specify the desired number of MPI processes and threads (see the reference manual).

**Algorithm 2:** Pseudocode of the hybrid MPI/multithreaded approach on each process (with id *myId*) to complete the biclusters.

1 INPUT only in Process 0: $m\times \frac{n}{32}$ 32-bit integer matrix *D* with the encoded data

2 INPUT only in Process 0: Set *S* with *nb* initialized biclusters

3 INPUT: Integer *mnr* with the minimum number of rows per bicluster

4 *MPI*_*Bcast* with *D* from Process 0 to the others

5 Calculate *myIniB* and *myLastB*

6 **if** *myId* == 0 **then**

7  Create *MPI*_*Window* *W* with $nb\dot{(}2+\frac{n}{32})$ 32-bit integers accessible to all processes

8  **for** *Each bicluster b in position j of S* **do**

9   Copy the id of the first attribute of *b* in $W[\left(2+\frac{n}{32}\right)\cdot j]$

10  Copy the id of the second attribute of *b* in $W[\left(2+\frac{n}{32}\right)\cdot j+1]$

11  Copy the $\frac{n}{32}$ integers of the pattern of *b* in $W[\left(2+\frac{n}{32}\right)\cdot j+2]$

   **end**

12 *MPI*_*Win*_*fence* to guarantee that copies to *W* are completed

  **end**

  **else**

13 *MPI*_*Win*_*fence* to guarantee that the necessary initial bicluster information is in *W*

14 Get the information of *W* from $myIniB\dot{(}2+\frac{n}{32})$ to $myIniB\dot{(}2+\frac{n}{32})$

15 Create a list with the information copied from *W* in the previous line

  **end**

16 # Multiple threads responsible of different biclusters

17 **for** *Each bicluster b from myIniB to myLastB* **do**

18 *nr* ≔ 2

19 **for** *Each row i from* 0 *to m* **do**

20  **if** *i is not one of the initial row ids of b* **then**

21   *p* equal to the pattern of *b*

22   *j* ≔ 0

23   **while** (*j* < *n*) ∧ (*p*[*j*] == *p*[*j*] ∧ *D*[*i*][*j*]) **do**

24    *j* ≔ *j* + 1

    **end**

25   **if** *j* == *n* **then**

26    Insert row *i* as part of *b*

27    *nr* ≔ *nr* + 1

     **end**

     **end**

   **end**

28 **if** *nr* < *mnr* **then**

29  Remove *b* from the list of biclusters

   **end**

  **end**

**Fig 2:**
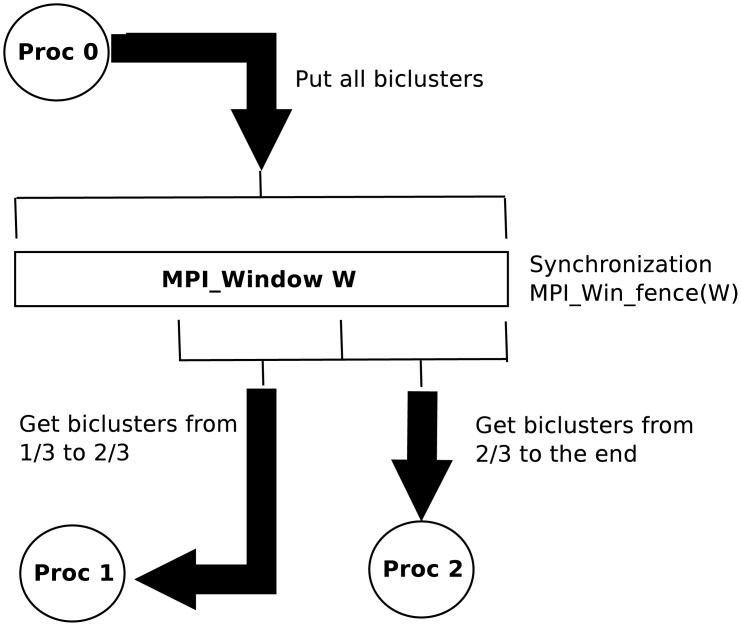
Example of a bicluster distribution for a program with three MPI processes using RMA windows. The main process (Proc. 0) copies the whole data to the window and, after a synchronization that guarantees that the copies have been completed, the other processes directly get only the information of their associated biclusters.

Similarly to the encoded data, at the beginning of the phase the information of the initialized bicluster structures is only available on the main process memory. In this case it must be distributed (not replicated) among all processes so that each one only saves the information of those biclusters that it will work with. It is performed with one-sided RMA routines which in general are more efficient than two-sided counterparts, as mentioned in the MPI background subsection. RMA communications work with windows, i.e., arrays of data that belong to one process but are directly accessible to the other, without requiring any synchronization between senders and receivers. This window is created in the memory of the main process (Line 7) with enough space to store all the initial information of the bicluster structures: the id of the two rows that are already included as well as the $\frac{n}{32})$ integer pattern. The information of each bicluster is consecutively stored in the window (Lines 8-11). Then, each process accesses to their associated data just with one get routine (Line 14) after a synchronization that guarantees that the data has been effectively copied to the window (Lines 12-13). Fig 2 illustrates with an example the procedure of the bicluster distribution.

Once all processes have the necessary information, each one can start the completion of its associated biclusters by launching several threads (each thread responsible of different biclusters). No synchronization is needed among processes or threads as the procedure is completely independent among biclusters. Assume that the initial pair of rows of a certain bicluster is (*r*_1_, *r*_2_). Then we must analyze all the rows *r*_*x*_ different than *r*_1_ and *r*_2_ (loop between Lines 19 and 27). As explained for *BiBit* a row must be included in the bicluster when the result of the logical AND operations between the bicluster pattern and the row data are the same (Lines 23-26). Every time that *ParBiBit* adds a new row it updates the variable *nr* with the number of rows per bicluster (Line 27). Once all rows different than *r*_1_ and *r*_2_ have been tested, we remove the bicluster from the list if the number of rows *nr* is lower than *mnr* (Lines 28-29). Finally, each process prints the information of their associated biclusters into the output file.

## Experimental results

Two Intel platforms with different characteristics are used to evaluate the efficiency and scalability of *ParBiBit*, as well as to compare its performance to the original *BiBit*. Table 1 summarizes their characteristics. Both GNU compilers support the C++11 standard, and all the experiments are compiled with the -O3 flag. The evaluation shown in this section is focused on performance in terms of execution time, as the biclustering approach of *ParBiBit* is the same as in *BiBit* and its accuracy was already satisfactorily tested in previous studies [9]. The input datasets were created by randomly generating one and zero values. We vary the number of samples (100 and 200), the number of attributes (12,800, 25,600 and 51,200), and the percentage of one values (10% and 15%). The percentage of one values has significant influence on the speed as different amount of biclusters are found for the same dataset dimensions. The more biclusters are found with not repeated pattern, the more analyses must be made in the computationally intensive bicluster completion phase of the algorithm. The results shown in this section were obtained by searching for biclusters with at least 2 samples and 1% of the attribtes (i.e., 128, 256 and 512 for the datasets with 12,800, 25,600 and 51,200 attributes, respectively).

**Table 1:** Characteristics of the test platforms used in the experimental evaluation.

	Platform1	Platform2
Nodes	8	8
CPU type	Intel Sandy Bridge	Intel Haswell
CPUs per node	2	2
Cores per CPU	8	12
Clock frequency	2.20GHz	2.50GHz
Memory per node	64GB	128GB
Network	InfiniBand FDR	
MPI Compiler	Open MPI 1.7.2	
C++ Compiler	GNU 4.9.2	GNU 5.3.0

**Fig 3:**
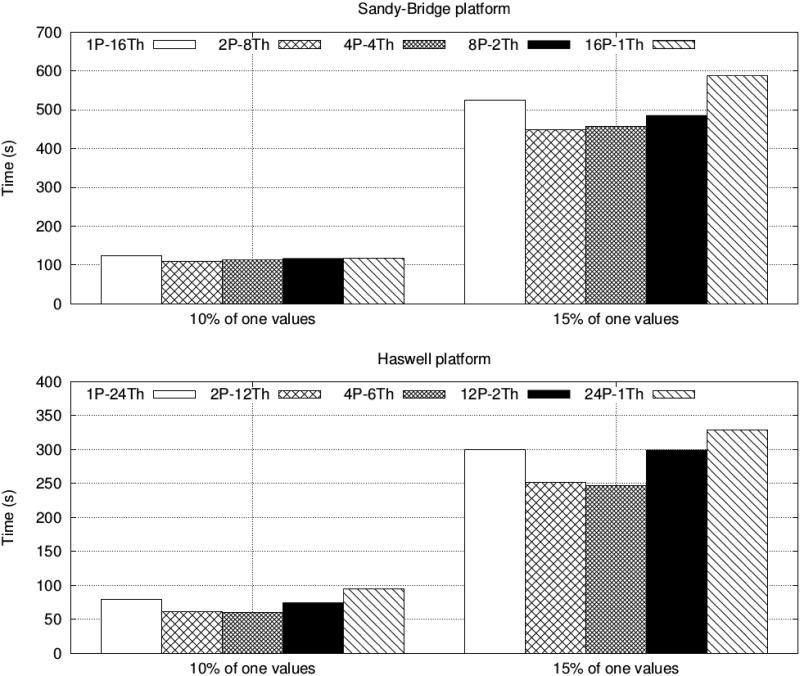
Runtime of *ParBiBit* on one node of each system with different configurations of processes and threads when searching biclusters on the datasets with 12,800 attributes and 200 samples.

**Fig 4:**
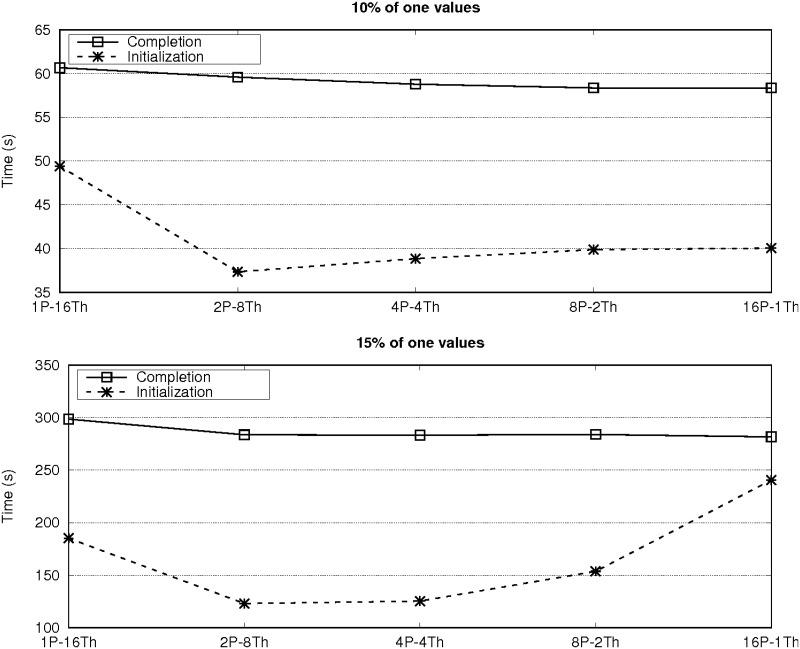
Partial time of the bicluster initialization and completion steps on one node of the Sandy Bridge system for different configurations of processes and threads. *ParBiBit* searches for biclusters on the datasets with 12,800 attributes and 200 samples.

**Fig 5:**
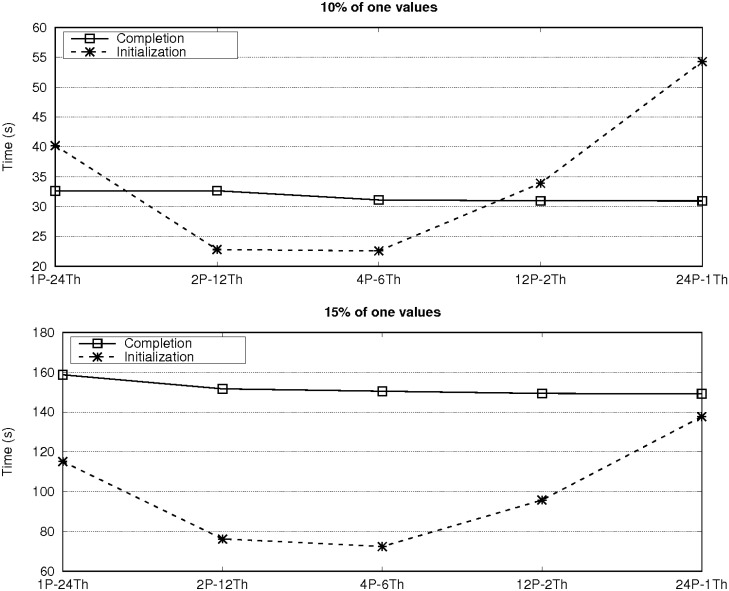
Partial time of the bicluster initialization and completion steps on one node of the Haswell system for different configurations of processes and threads. *ParBiBit* searches for biclusters on the datasets with 12,800 attributes and 200 samples.

The experimental evaluation started by finding the best configuration of the number of threads and MPI processes for *ParBiBit* on each system. Fig 3 shows the runtime on a single node of each platform (16 and 24 cores on the Sandy Bridge and Haswell systems, respectively) for different configurations. The datasets with 12,800 attributes and 200 samples with both 10% and 15% of one values are used in this case as illustrative examples. An intermediate configuration is the best option on both platforms: two processes and eight threads on the Sandy Bridge system, while four processes and six threads on the second machine. The performance differences are mainly generated during the phase of biclustering initialization as we must find a balance between using more threads to increase parallelization in this step (remind that only one process is used to do so) and reducing the number of threads to limit the overhead due to mutex synchronization. During the biclustering completion, increasing the number of MPI processes leads to more communication operations for the encoded data replication and the initial bicluster distribution. Nevertheless, the increase of communication overhead is not significant compared to the total runtime of this phase. Figs 4 and 5 show the partial runtime of the initialization and completion steps for the different configurations on each platform to illustrate the previous assertions. As a rule of thumb, using as many processes as CPUs per node and as many threads as cores per CPU is a good starting point for achieving optimal or quasi-optimal performance on most systems. From now on all the experimental results shown in this manuscript were obtained with the best configuration for each platform.

Table 2 shows a comparison of the runtime (in minutes) of the original *BiBit* tool and *ParBiBit* using different amount of cores and nodes. The first conclusion that can be obtained is that *ParBiBit* is significantly faster than *BiBit* even when using the same amount of resources (one core). In fact, *ParBiBit* is on average 2.70 and 3.35 times faster on the Sandy Bridge and Haswell platforms, respectively. Furthermore, *ParBiBit* is able to complete the biclustering of the largest dataset (51,200 rows, 200 columns and 15% of one values) while *BiBit* was not able to finish in the maximum computation time allowed to the users on the clusters (four days). Two are the reasons for this performance improvement on one core: 1) a more efficient memory and I/O management of our C++ implementation compared to the Java one of *BiBit*; and 2) the use of 32-bit logical AND operations for the encoding data instead of being based on 16-bit integers.

**Table 2:** Runtimes (in minutes) of *ParBiBit* using up to eight nodes with the best configuration of threads and MPI processes per platform. The runtime of the sequential *BiBit* tool are also included for comparison purposes. Both tools look for biclusters with at least 2 samples and 1% of the attributes present in the input dataset. − means that *BiBit* was not able to finish the biclustering in the maximum time allowed for computation (four days).

Platform	Att.	Sam.	% of ones	Bicl.	*BiBit*	*ParBiBit*		
						1 core	1 node	8 nodes
Sandy Br.	12800	100	10	2359	7.41	5.51	0.48	0.39
			15	4950	29.48	11.50	1.37	0.79
		200	10	9510	49.88	18.50	1.82	0.96
			15	19900	214.27	73.64	7.49	3.31
	25600	100	10	2313	40.52	24.25	2.02	1.44
			15	4950	159.18	59.70	5.87	2.75
		200	10	9357	243.65	117.99	9.23	3.96
			15	19900	1541.89	505.57	43.88	14.19
	51200	100	10	2283	249.19	101.23	7.62	5.67
			15	4950	1069.27	318.97	31.79	11.36
		200	10	9855	2354.67	474.06	41.21	14.63
			15	20137	-	1540.39	118.40	31.16
Haswell	12800	100	10	2359	5.58	1.62	0.45	0.34
			15	4950	28.07	10.54	0.85	0.47
		200	10	9510	51.59	12.50	1.00	0.54
			15	19900	200.97	58.06	4.12	1.91
	25600	100	10	2313	28.31	7.97	1.58	1.29
			15	4950	150.80	56.25	3.90	1.88
		200	10	9357	313.16	100.11	5.19	2.16
			15	19900	1564.62	531.15	25.04	8.73
	51200	100	10	2283	103.18	36.16	6.22	5.02
			15	4950	1123.99	311.25	17.38	7.74
		200	10	9855	1869.82	424.97	27.67	9.21
			15	20137	-	1660.45	94.53	29.71

**Fig 6:**
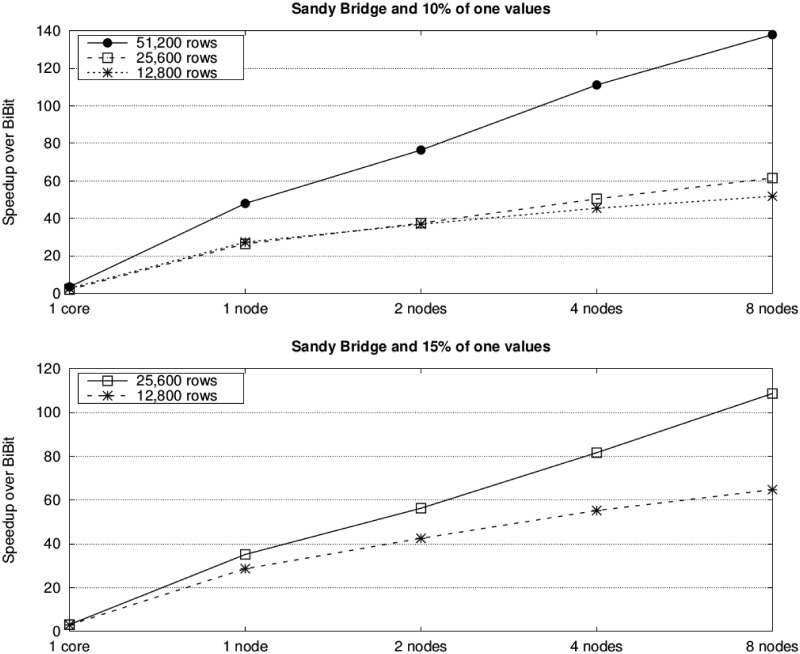
Speedups of *ParBiBit* over *BiBit* for varying number of nodes on the Sandy Bridge platform. Each line represents a different number of attributes (the number of samples is fixed to 200) and each graph is associated to a different percentage of one values.

**Fig 7:**
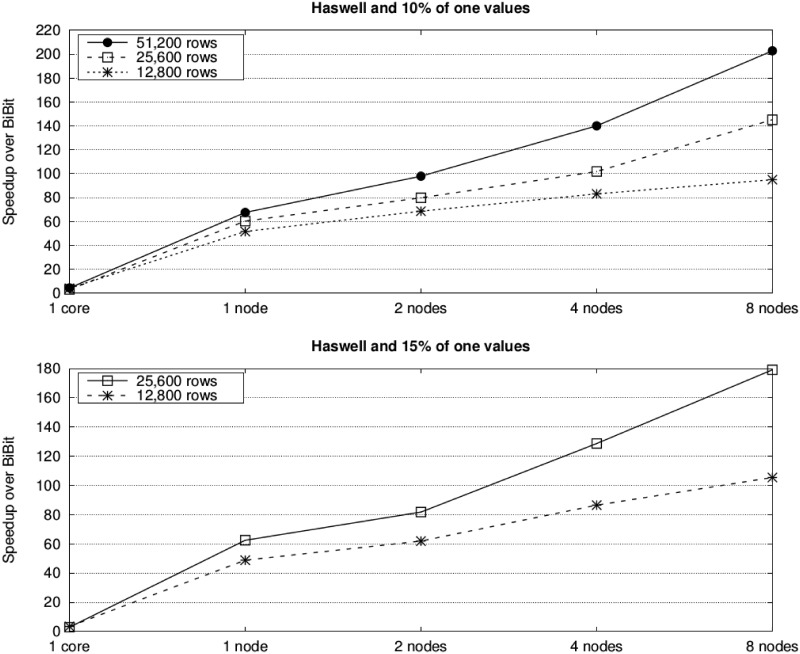
Speedups of *ParBiBit* over *BiBit* for varying number of nodes on the Haswell platform. Each line represents a different number of attributes (the number of samples is fixed to 200) and each graph is associated to a different percentage of one values.

**Fig 8:**
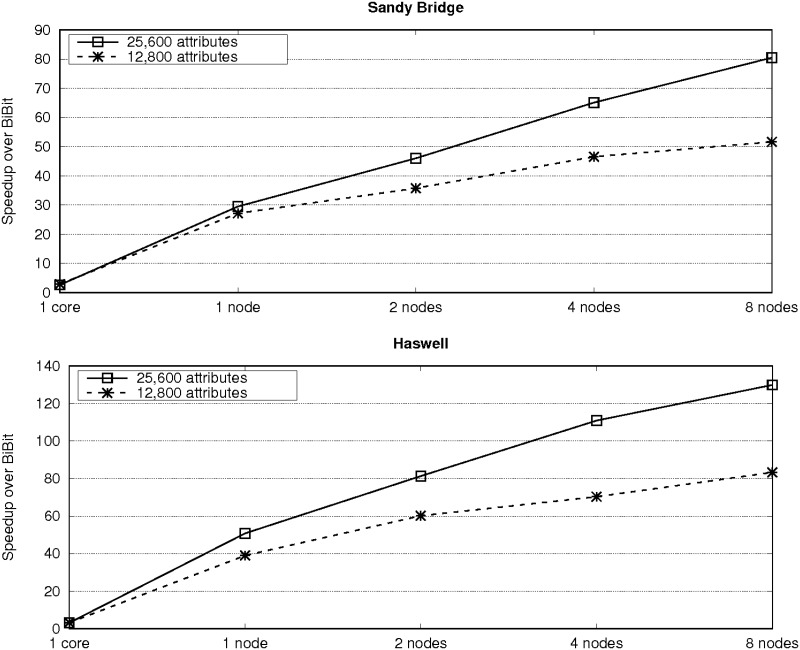
Speedups of *ParBiBit* over *BiBit* for varying number of nodes. Each line represents a different number of attributes (the number of samples is fixed to 100, while the percentage of one values is 40%).

Furthermore, the use of our two-level parallelization on a multicore cluster significantly reduces runtimes. For instance, *BiBit* needs more than four days to process the dataset with 51,200 attributes, 200 samples and 15% of one values, while *ParBiBit* reduces the runtime to only around 94 and 30 minutes on one and eight nodes of the Haswell system, respectively. Figs 6 and 7 provide an insight of the benefit in terms of performance that can be obtained by our tool compared to the state of the art. As expected, the acceleration is higher on the Haswell platform as it provides more resources (24 instead of 16 cores per node). In an attempt to show the adequacy of *ParBiBit* to different scenarios, experiments with 40% of one values and selecting 20 samples per bicluster have also been executed. For simplicity, acceleration is represented in Fig 8. It is worth noting that this paper does not include a comparison to other parallel tools because, as mentioned in the related work section, up to our knowledge there is no publicly available tool to accelerate the biclustering procedure of binary data on parallel architectures.

It was not possible to include in these figures the speedups over *BiBit* for the most computationally expensive dataset. As mentioned before, the original Java tool did not finish in four days (the maximum allowed runtime of the systems). Instead, Table 3 shows the scalability of our tool for this dataset using as baseline the runtime of *ParBiBit* on only one core. These results (and the ones in Figs 6 and 7) prove that our tool scales in all scenarios at least up to eight nodes. In our opinion, the parallel approach included in *ParBiBit* provides good scalability. Its main strength is that we focused on obtaining very high performance during the most computationally demanding phase with the hybrid MPI/multithreaded parallelization: more than 90% of parallel efficiency during bicluster completion even using the eight nodes. As drawbacks we should mention that some parts cannot be parallelized (I/O routines, data encoding), the bicluster initialization is only parallelized with threads, and some communications (with their associated overhead) are compulsory.

**Table 3:** Speedups of *ParBiBit* for varying number of nodes using as baseline the runtime on a single core. The input dataset contains 51,200 attributes, 200 samples and 15% of one values.

Num. Nodes	Sandy Bridge	Haswell
1	13.01	17.57
2	21.66	26.40
4	35.35	46.12
8	49.44	55.88

Finally, the acceleration was also tested in a scenario with non-binary real data. Authors in [12] explain a method to work with data that is not binary: 1) standardize the data, generating a real value matrix with a mean of zero and a variance of one; 2) data discretization to establish 12 different levels of gene expression values; and 3) execution of *BiBit* or *ParBiBit* over this dataset by applying the optional binarization step (point 2 in “Methods”). In order to provide a fair comparison with *BiBit* the same real dataset used in its manuscript [12] for this last performance evaluation (i.e., a central nervous system embryonic tumor gene expression dataset obtained from DNA microarray technology [36] with 40 tumor samples and 7,129 genes). The runtime of *BiBit* to complete the biclustering of the 11 binary matrices that are generated by this real dataset on both platforms is higher than seven hours. Each of the 11 matrices has a different percentage of one values. ParBiBit is also beneficial for this real dataset as it reduces the runtimes to less than two minutes.

## Conclusions

Current biclustering data mining algorithms allow to extract useful biclusters from large binary datasets. Even though these algorithms can provide highly accurate results, the procedure of extracting those biclusters is a very time-consuming task, which can represent a significant performance bottleneck in some fields such as gene expression data analyses. To overcome this issue, we propose to take advantage of parallel architectures as the modern distributed-memory systems to alleviate this runtime bottleneck, thus being able to process very large binary datasets within reasonable times.

In this paper we introduce *ParBiBit*, a parallel biclustering tool that significantly speeds up the procedure of discovering interesting biclusters from binary data. Our tool benefits from a two-level parallelism strategy by combining message-passing with multithreading in order to fully exploit the computing resources of multicore CPU clusters. This hybrid approach has been evaluated on two representative systems, showing experimental evidence of significant performance improvements when compared with the original *BiBit* tool. In fact, *ParBiBit* reduces the execution time of *BiBit* by up to 203x when processing a dataset with 51,200 attributes on a 8-node Intel Haswell-based cluster. The experimental results also indicate that our tool provides good scalability. The source code of the parallel tool described in this paper is distributed as free software, being publicly available under an open-source license at https://sourceforge.net/projects/parbibit/.

As future work, we aim to adapting *ParBiBit* to exploit other parallel architectures such as GPUs and/or Intel Xeon Phi coprocessors, as well as developing a counterpart to run non-binary biclustering on multicore clusters.
